# Depression, reduced education, and bias perceptions as risk factors of beliefs in misinformation

**DOI:** 10.1038/s41598-022-20640-7

**Published:** 2022-09-30

**Authors:** Marco Delmastro, Marinella Paciello

**Affiliations:** 1 Ca’ Foscari University, Venice, Italy; 2 Enrico Fermi Research Center, Rome, Italy; 3 Uninettuno Telematic International University, Corso Vittorio Emanuele II, 39, 00186 Rome, Italy

**Keywords:** Psychology, Health care

## Abstract

The spread of misinformation and conspiracy theories related to COVID‐19 has represented one of the several undesirable effects of the current pandemic. In understanding why people can be more or less at risk to believe in misinformation, emotional distress and education could play a crucial role. The present study aims to analyze the relationship among depressive symptoms, education, and beliefs in misinformation about COVID-19 during the early phase of the pandemic. We do this through a cross-sectional study carried out on a random and representative sample of the Italian population that allows us to go and verify the co-evolution of many factors: i.e., beliefs in misinformation, symptoms of depression, perceptions about COVID-19, ways in which citizens got informed about the pandemic, and sociodemographic characteristics (e.g., age, gender, education). The results show that the relationship between depression and beliefs in misinformation exists and is more complex than hypothesized because it is mediated by individual perceptions. In particular, the most at-risk people to believe in misinformation show higher bias perceptions, higher depression, and lower education. Practical implications are discussed suggesting a supportive intervention at both individual and social levels.

## Introduction

In 1964, in a seminal paper, Richard Hofstadter defined belief in conspiracy theories (herein also CTs) as “*a style of mind*”, a “*paranoid style*”. In essence, his view was that this style of mind was “*a persistent psychic phenomenon, more or less constantly affecting a modest minority of the population*”^1^. This approach has influenced the subsequent debate by relegating those who believe in conspiracy theories and, more generally, misinformation to a small group of paranoids^2^. Recent surveys^3^ show on the contrary that believing in conspiracy theories and misinformation is widespread among the world population and covers the most disparate issues from vaccines to aliens, from politics to climate change. On the other hand, the persistence in time of the phenomenon, confirmed by historical anecdotes^2^ and some longitudinal studies^4^, highlights that beliefs in false narratives are a structural phenomenon, typical of all popular cultures.

In this article, we aim to contribute to the scientific debate by examining the role of depression, education, and biased perceptions in believing in “*misinformation and conspiracy theories related to COVID‐19, which is likely one of the most significant pandemics of our lifetime*”^5^.

The focus on emotional distress (i.e., depressive symptoms) is because the outbreak of the COVID-19 pandemic has constituted a major threat to physical and mental health^6,7^. In particular, the current pandemic has exacerbated depressive symptoms^8^, and it has questioned one's own personal control over life outcomes. In addition, it has exacerbated the effect of situations of physical isolation (partly because of the adopted distancing and lockdown restrictions taken by governments to contrast the pandemic) that are associated with depressive symptoms^9^ and the beliefs of citizens. This emotional distress, by interacting with cognitive and social factors can facilitate the spread of conspiracy beliefs and more generally misinformation narratives during persistent stressful COVID-19 conditions^10,11^.

The relationship between emotional distress and beliefs in false narratives may be related to biased perceptions about COVID-19^12^. Indeed, depressive feelings and a sense of vulnerability can lead citizens not to fully perceive a new and complex phenomenon whose knowledge has changed drastically and quickly over time. In psychological literature^13^, several studies have already begun to assess the effect exerted by negative emotions exacerbating paranoid thinking, in both clinical and non-clinical populations^14^. Emotional distress such as depression, irritation, or anxiety could increase the self-perception of vulnerability, especially under very stressful conditions (e.g., major life events, existential threats). The self-perception of being low-power is associated not only with depression but also with specific information-processing biases such as “jumping to conclusions” in the face of probabilistic information and misunderstanding the intentions of others^15^.

Moreover, misinformation finds a fertile environment where COVID-19 is not properly understood by many citizens within a cognitive framework based on trusted scientific findings. In line with this perspective, in a seminal paper, Abalakina-Paap et al. showed that “*beliefs in conspiracies are related to feelings of alienation, powerlessness, hostility, and being disadvantaged. There was no support for the idea that people believe in conspiracies because they provide simplified explanations of complex events*”^16^. Much depends on how citizens acquire information on COVID-19^10^. Previous findings show that conspiracy thinking may be associated with avoidance of mainstream news media and with a tendency to acquire news and information mainly through alternative news sources, such as those shared via social media^17^. In addition, algorithms in social media platforms can lead to echo chambers and confirmation bias among users, which is a main issue in believing in misinformation and fake news^18,19^. On the other hand, scientific and institutional sources have become established among part of the population at this stage and many citizens have started to consult directly to acquire accredited and validated news and information on the epidemic and its development.

In this context, it is also useful to check whether high levels of education can help to disengage false beliefs, which as mentioned in the case of health phenomena can have very undesirable effects on public health and social welfare. Previous studies show that a stronger belief in conspiracy theories is significantly associated with lower analytic thinking and greater intuitive thinking^20^. To do so, educational attainment may be used as a proxy for individuals' analytical reasoning. This has two undoubted benefits (and of course some drawbacks^21^). First, educational attainment offers an objective element that, although it varies across geographic contexts (but this issue is less prominent in the case of an analysis conducted in a single nation), is less volatile than ad-hoc tests. In addition, the educational system, including permanent education, is a “variable” on which governments can and should operate. Overall, it is important to check whether the worsening of the depressive symptoms due to the pandemic is also correlated with the greater gullibility of individuals towards misinformation narratives, considering different levels of education.

All these factors—depressive symptoms, education levels, and ways in which news is acquired—can facilitate bias perceptions. It is therefore interesting to verify to what extent beliefs in misinformation and conspiracy theories (about COVID-19) are associated with a bias in the perception of the phenomenon and/or by a more adherent (or on the contrary skeptical) attitude towards them.

To do so, we conducted a cross-sectional study on a random and representative sample of the Italian population (aged 16+) right after the first lockdown phase (i.e., in June 2020), to collect the immediate reactions of citizens to the emergency. Indeed, we carried out the survey as soon as the lockdown phase was just over (i.e., so-called “Phase 1”), with the re-opening of manufacturing industries and construction sites and the restart of movements across Italian regions (from the 3rd of June). This timeframe appears ideal to conduct a field analysis because the whole lockdown phase had just ended, the citizens' memory of the period just passed was still intact and the organization of such a complex survey was feasible.

## Method

### Design and procedure

We collected data through an online survey among a sample of the Italian population aged 16+ (see the Supplementary Material for a detailed description of the study design). The design comprehends a wide range of aspects, from depressive symptoms to the perception of COVID-19 and assessment over news, real and false, circulating online on the epidemic. Sociodemographic characteristics were also assessed. The survey contained closed-ended questions only and lasted an average of 20 min per participant.

The survey was fielded from June 4, 2020, to June 19, 2020.

In this context, the interviewees were informed that all their personal data would be acquired anonymously and in full compliance with privacy laws.

All methods were carried out following the Declaration of Helsinki. The study has been conducted in accordance with Enrico Fermi Research Center ethical guidelines and was reviewed and validated by the Enrico Fermi Research Center internal board. Informed consent was obtained from all participants and a parent and/or legal guardian for participants under 18.

Data analysis was performed using Stata Statistical Software and R.

### Sample characteristics

Recruitment and data collection were carried out by a polling firm, to ensure the construction of a random and representative sample of the Italian population (16+). Participants were randomly recruited from census panels. Significant efforts were made to maximize the representativeness of the sample by using software generating representative samples of the population and by including hard-to-reach groups through targeted recruitment.

The final sample was composed of 4972 Italian individuals, representative of the Italian population in terms of age, gender, and geographical area. More specifically, the sample design and the stratification were based on the following variables: (i) age (7 age groups: 16–17; 18–24; 25–34; 35–44; 45–54; 55–64: 65+); (ii) gender, (iii) geographical breakdown (all Italian regions and size of the residential community; 7 classes), (iv) education (2 classes: graduates and non-graduates).

All responses were anonymized to fully respect the privacy of participants.

### Measures

The measures introduced in the analysis concern the following aspects: beliefs of the participants in misinformation on COVID-19 (and CTs); type of news sources used by participants to get informed about the pandemic; psychological condition of participants related in particular to depression; socio-demographic characteristics (including territorial control variables relating to the area in which the participants live). In addition, given the nature of the phenomenon under investigation, information has been acquired about the either direct (i.e., personal) or indirect (in the family) experience of participants of COVID-19 disease.

To measure these aspects, a multidisciplinary research approach has been adopted^22,23^, including tools ranging from psychology (for the assessment of mood and feelings), sociology (for the news sources and socio-demographic characteristics), and behavioural science (for beliefs in misinformation and perceptions about COVID-19).

#### Beliefs in misinformation (and conspiracy theories) about COVID-19

A direct approach has been chosen to assess the gullibility of individuals with misinformation and conspiracy theories^24^ (on the different types of false news narratives and their classifications see[^22^, Ch. 2], on conspiracy theories and their definition see^2,4^). The participants were presented with 6 news about the COVID-19 spread on the web, of which 3 were real and 3 false (note that some of the fake news belongs to conspiracy theories circulating about the origin of the coronavirus, see below). The news was considered false based on an assessment by third-party independent fact-checkers (i.e., Facta, Newsguard, and Pagella Politica). The news was randomly extracted from 12 news reports, without the participants knowing if and how many were real or false. The participants had to assess for each news if it was either false or real. Consequently, we can measure beliefs in misinformation by checking, for any false news, whether the individual has correctly assessed its degree of reliability.

#### COVID-19 information sources

Participants were asked about the sources of information they used to get informed about COVID-19. The questions concerned all major sources of information (traditional, algorithmic, and non-traditional), including institutional sources (such as the Ministry of Health, the Department of Civil Protection, and the World Health Organisation) that in the pandemic period have seen an exponential growth of direct contacts by citizens.

#### Health: COVID-19 and depressive states

For this research, the first health factor concerns direct experience with COVID-19. This aspect is essential in understanding the cognitive dynamics of citizens compared to the phenomenon under investigation (i.e., beliefs in misinformation about COVID-19). So, participants were asked whether from the beginning of the pandemic (at the time of the survey 4 months had passed since the outbreak of the virus in Italy: i.e., the virus was first confirmed to have spread to Italy on January 31, 2020, when two Chinese tourists in Rome tested positive for the virus) they or their family members had been negatively affected by COVID-19.

To evaluate the depressive feelings of individuals a psychometric approach was adopted. Due to the special nature of the period which prevented meeting participants face-to-face, a psychometric self-reporting methodology was chosen. We adopted the short version of the Mood and Feelings Questionnaire (SMFQ)^25^ which includes 13 items indicating how much individuals have felt or acted depressed during the last few weeks (e.g., “I felt miserable or unhappy”, “I felt lonely”). The answers are given on a three-point scale where respondents are asked to decide if the statements are “true”, “sometimes true”, or “not true”. Scoring of the SMFQ is obtained by summing together the point values of responses for each item. The response choices and their designated point values are as follows: “not true” = 0 points, “sometimes true” = 1 point, “true” = 2 points. Higher scores on the SMFQ indicate more severe depressive symptoms. The range of scores on the SMFQ varies from 0 to 26. A score of 12 or higher may indicate the presence of depression in the respondent^9,26^.

#### Socio-demographic characteristics

Respondents were asked to indicate age (in terms of the number of years since birth). Gender was measured with “female” used as the control category in the estimates (see below). Information regarding educational attainment (i.e., the highest level of education that a person has successfully completed) was codified as follows: 0 = no degree; 1 = elementary education, 2 = middle school; 3 = high school; 4 = graduation; 5 = master’s degree/Ph.D. A dummy variable has also been introduced which takes the value of 1 when the individual lives alone (it is 0 otherwise). Finally, geographical control variables (see Table A.2 in the Supplementary Material) have been introduced that measure the size of the municipality in which the individual lives and the spread of COVID-19 in the same area at the time of the survey (31 May 2020).

#### Perception of COVID-19

A final aspect concerns the perceptions of the participants about COVID-19^27^. To do this, the participants were given 5 multiple choice questions regarding the main features of COVID-19 known at the time of the survey. Accordingly, an indicator of bias perceptions has been constructed that takes value 1 (maximum) in the case of all wrong answers, is equal to 0 (minimum) in the opposite case in which participants have successfully answered all questions, and is between 0 and 1 in all other cases.

## Results

As a first step, we analyze the correlation between beliefs in misinformation and three sets of factors: the socio-demographic characteristics of individuals, how they got informed (about COVID-19), and the state of health (see Table A.1 in the Supplementary Material).

From a statistical point of view, an econometric analysis was performed that allows all factors to be considered together (including geographic control variables and those related to individual news items – i.e., type and diffusion). Since each participant was administered 6 news items on COVID-19, 3 of which were false (see the Method and SM), the model used is a probit (in which the dependent is 1 if the individual believes a false news item and 0 otherwise) panel data (with the number of observations equal to the responding individuals times 3, i.e., the number of false news items submitted to each participant). Results are presented in Table 1 (model I).

**Table 1:** Result of probit panel data models.

Categories	Model	(1)	(2)
	Variables	Belief in fake news	
Socio-demo	Gender (female = 1)	0.0551*	0.0748**
		(0.0311)	(0.0306)
Socio-demo	Age (number of years)	0.000178	− 0.000957
		(0.000979)	(0.000963)
Socio-demo	Living alone (= 1)	0.0935**	0.106**
		(0.0450)	(0.0444)
Socio-demo	Education (6 degrees, increasing order)	− 0.0712***	− 0.0586***
		(0.0196)	(0.0192)
News about COVID-19	Number of news sources (0–22)	− 0.0105**	− 0.00547
		(0.00434)	(0.00424)
News about COVID-19	Non-traditional news outlets (= 1)	0.257***	0.137***
	(traditional sources = benchmark)	(0.0526)	(0.0522)
News about COVID-19	Algorithmic news sources (= 1)	0.115**	0.0137
	(traditional sources = benchmark)	(0.0582)	(0.0580)
News about COVID-19	Institutional news sources (= 1)	− 0.117***	− 0.0770**
	(traditional sources = benchmark)	(0.0329)	(0.0323)
Perception about COVID-19	Bias in perception		0.899***
	(index: 0–1)		(0.0739)
Health	Mood (SMQF, 0–26)	0.00808**	0.00129
	(> 11 clinical depression)	(0.00323)	(0.00319)
Health	COVID-19 in family (= 1)	0.138**	0.0655
		(0.0609)	(0.0594)
	Constant	0.119	− 0.0905
		(0.137)	(0.135)
	Geographic controls	YES	YES
	News controls	YES	YES
	Observations	13,572	13,572
	Number of individuals	4524	4524

*The estimates in the table refer to probit panel data models, with robust standard errors. The dependent variable is 1 when the individual erroneously believes in fake news. For each variable, the coefficient, the standard error (in parentheses), and the level of significance are reported as follows: *** significant at 99%; ** significant at 95%.

Interestingly, all else being equal, people who live alone are significantly (at 95%) more likely to believe fake news. This relationship shows how false beliefs are most associated with situations of physical isolation, which became dramatically more substantial during the pandemic (with the measures taken by governments to contrast the spread of the virus that led Italy to lockdown restrictions during the first phase of the outbreak).

Conversely, educational attainment emerges as a decisive factor (99% significant) in explaining non-beliefs in misinformation. Reduced education appears to be a significant risk factor for gullibility in misinformation.

How individuals get informed is highly correlated to their likelihood to believe in misinformation. First, the more individuals consult different sources, the less likely they are to believe fake news. This indicates how much access to a diversity of viewpoints can develop a critical attitude in the individual. Second, the specific type of (primary) source of news and information about COVID-19 is significantly correlated to individuals' beliefs: people who use alternative, non-accredited sources tend to adhere more readily to narratives of misinformation. An interesting aspect is the widespread direct access of citizens to institutional sources providing information and data about COVID-19 (as many as 35.9% of the Italian population used institutional sources as their first source of information, see Table A.3 in SM). This active attitude, not mediated by journalistic activity, is negatively associated with individuals' tendency to believe in false narratives (the coefficient is negative and significant at 99%).

Turning to emotional distress, people who developed symptoms of depression appear, with a significantly higher probability, to believe in misinformation. Likely, these negative states could increase beliefs in misinformation because they could confirm the depressive-related ideas that events are uncontrollable, and individuals are vulnerable and powerless.

Looking at the health issues, it may seem counterintuitive the result of the COVID-19 variable whose coefficient is positive and significant (at 95%). One might have expected that those who have already experienced the disease (themselves or their families) have more information available to them and thus are less likely to believe in false narratives. This result applies in the case of contagions in the same area where the individual lives: i.e., beliefs in fake news are negatively (and significantly) correlated with the spread of contagion in the area where citizens live (i.e., geographical control variables, see the Supplementary Material). On the contrary, on an individual level, these negative health experiences, which are associated with depressive feelings, may have increased beliefs in misinformation because they confirm the perception of powerlessness in dealing with so harmful events. However, given that available data (i.e., cross-sectional) the estimates do not allow to identify causal relations, so that the opposite relationship may have prevailed: i.e., individuals who believe in misinformation narratives are less able to manage Covid-related events and expose themselves more to the dangers of disease contagion.

We have then proceeded to estimate the value of the probability of believing in misinformation about COVID-19 for different values of the significant explanatory variables (Table 2). Note that although the correlational nature of the exercise requires that the estimates should be taken with some caution, given that the sample is random and representative of the whole population, these can be considered as estimates of the entire Italian population (16+). This probability decreases from 50 to 38% going up the level of educational attainment. In contrast, the probability increases from 39 to 46% for increasing levels of depression. The size of the variation in the case of different news media mixes is also significant, in terms of both the number of sources used to get informed about COVID-19 (from 46 to 38% for an increasing number of sources) and the type of primary source (51% in the case of non-traditional sources and 39% in the case of institutional sources).

**Table 2:** Probabilities of believing in misinformation for different categories of citizens.

		Probability of believing in misinformation (%)
	Benchmark case*	42.5
Socio-demo	Living alone (= 1)	45.6
Socio-demo	Education min (no degree)	49.6
	Education mean (high school)	42.5
	Education max (post-grad)	37.9
News about COVID-19	Number of news sources: min (0)	45.6
	Number of news sources: mean (9)	42.5
	Number of news sources: max (22)	38.1
News about COVID-19	Traditional news sources	42.5
	Non-traditional news sources	51.0
	Institutional news sources	38.7
Health	Good mood (SMFQ = 0)	39.4
	Mood just above threeshold (SMFQ = 12)	42.5
	Maximum depression (SMFQ = 26)	46.2
Health	Covid-19 (= 1)	47.1

*The estimates in the table refer to a probit panel data model (i.e., model I Table 1), with robust standard errors. The benchmark case has been calculated as follows: average age (i.e., years = 49), living in a family, high-school education, average number of news sources on COVID-19 (i.e., 9), SMFQ index = 12, no COVID-19 in family.

Since the factors considered may vary jointly (e.g., those who live alone are more likely to be depressed^9^) we also compared three possible and opposite types of individuals: type A (i.e., people who are well-educated and well-informed, live in a family, and have a good mood), type B (i.e., people who are less educated, depend only on few non-established sources of news about COVID-19, live alone, and exhibit severe symptoms of depression), and the benchmark case (Table 3). The probability of believing in misinformation increases from a value of one-quarter (type A, 27%) to nearly three-quarters (type B, 71%).

**Table 3:** Probabilities of believing in misinformation for types of citizens.

Type of individual	Characteristics	Probability of believing in misinformation (%)
Type A	Max education, living in family, good mood, get informed from many sources	27.4
Benchmark	Average education, living in family, few symptoms of depression, got informed from few sources	42.5
Type B	No education, living alone, symptoms of depression, got informed from only non traditionional sources	71.3

*The estimates in the table refer to a probit panel data model (i.e., model I Table 1), with robust standard errors.

### Belief in CT and mood

Then we analyze only fake news that contains a conspiracy theory about COVID-19. In particular, it was submitted to 1,852 individuals a piece of news circulated online according to which “*The coronavirus strain that is spreading in China and abroad is a patented virus owned by an entity called The Pirbright Institute, partially funded by the Bill and Melinda Gates Foundation*”. This news appears to fully satisfy the definition of conspiracy theory according to which “conspiracy theories” are attempts to explain the ultimate causes of significant social and political events and circumstances with claims of secret plots by two or more powerful actors^2,28^.

The relationship between depressive feelings and beliefs in CTs is even more evident: those who have a normal mood (SFMQ index equal to 11 or less) believe only 13% of the time in a conspiracy theory, while this percentage rises to 33% for individuals with symptoms of depression (SFMQ index equal to 12 or more). This result is in line with the ‘poor me’ paranoid idea associated with depressive feelings and with also a misunderstanding of other intentions^15^.

**Figure 1:**
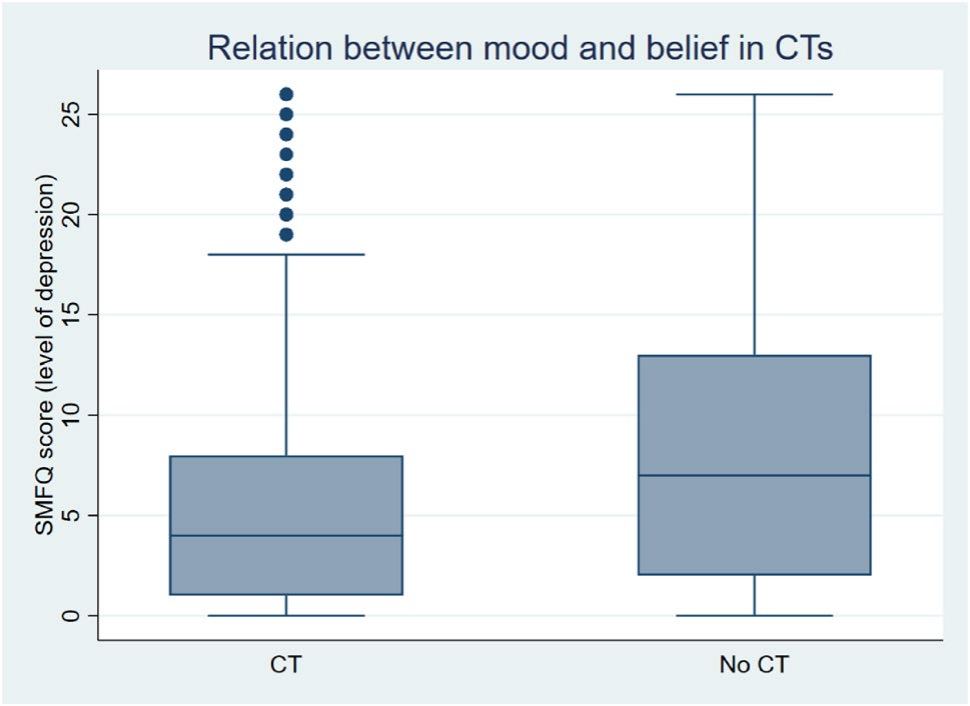
Boxplot of the mood (i.e., SMFQ index) for two categories of individuals: those who believe in a conspiracy theory about COVID-19 (CT) and those who don't (No CT).

Figure 1 shows the distribution of the SFMQ score for two categories of individuals: those who believe and those who do not believe in a conspiracy theory about COVID-19. From the boxplot it is evident that those who believe in a conspiracy theory display a significantly higher level of depression. Note that both the mean of the SMFQ index of the two groups (5.3 and 8.0) and the two distributions are statistically different at 99%, by a t-test and a Kolmogorov–Smirnov test, respectively.

### Bias in perception and belief in misinformation

The next step is to analyze whether (and how much) beliefs in misinformation narratives are related to a bias in the perception of COVID-19.

To do this, as explained above, participants were asked 5 multiple choice questions about the hitherto known characteristics of COVID-19. Based on the answers of participants, an index of the bias in perception of the phenomenon has been constructed, that is equal to 0 when the individual has guessed all the answers (maximum), is 1 when instead it has mistaken all the answers (minimum) and is between 0 and 1 in all other cases (see the Supplementary Material). We have included this variable in the previous probit panel data model. The results are shown in Table 1 (model II).

This variable, as expected, is very significant (at 99%) and positive, highlighting a strong correlation between bias perceptions and beliefs in misinformation (the Pearson correlation coefficient is 0.192 which is significantly greater than zero, *p* value = 0.00).

**Figure 2:**
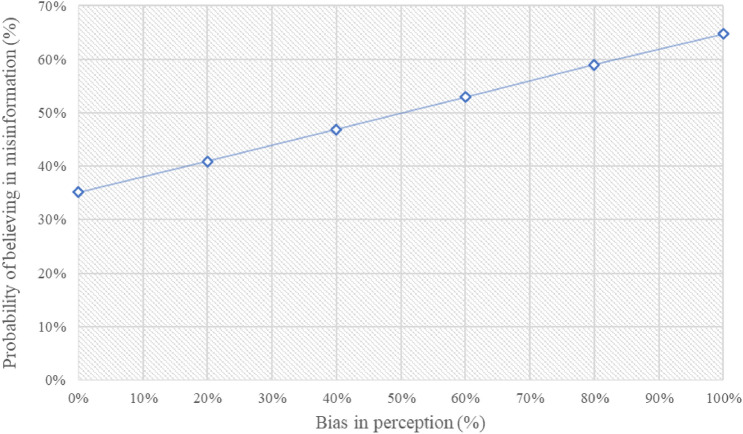
Relationship between bias in perception and probability in believing in misinformation. *Note The estimates in the figure refer to a probit panel data model (i.e., Model II of Table 1), with robust standard errors.

If we calculate the probability of believing in false narratives about COVID-19 depending on the bias in perception (Fig. 2), we observe that starting from a minimum level around 30% this probability grows to over 60% in the case of maximum bias, almost doubling its value.

Other interesting results are on the one hand the loss of explanatory value of the depression score and on the other the confirmation instead of the significance of educational attainment. In other words, depressive symptoms appear to be related to the beliefs of individuals only through bias perceptions.

## Discussion

Beliefs in misinformation (going as far as conspiracy theories) affect a large portion of the population and may be due to various reasons^12^. The article explored some factors that predispose individuals to be naturally more responsive and less skeptical about such false narratives. When regarding information about COVID-19 such beliefs assume crucial importance because they may induce behaviors dangerous for the individual and collective well‐being.

A relevant aspect is the relationship between emotional distress and beliefs in misinformation. In line with the literature on emotional distress and paranoid thinking^13,14,29^, results show that people who suffer from symptoms of depression are more likely to believe in misinformation about COVID-19. In the past few months, the psychological literature has shown how the pandemic has caused parts of the population to develop these symptoms^8^. Overall, the results suggest that there is a concrete risk of creating a social process characterized by a negative feedback loop: from the pandemic to depressive symptoms, that are associated with false beliefs about COVID-19, which in turn can be related to inappropriate behaviors that fuel the spread of the pandemic, and so on.

Nonetheless, we cannot exclude that also beliefs in misinformation may lead to increase in depressive mood. Indeed, consistently with the existential threat model^30^, conspiracy thinking can be a source of the existential threat itself. However, differently from the existing model, the present findings suggest that also depressive feelings (not only anxiety) could play a significant role in the case of existential threat events. Future longitudinal studies need to understand how depression could interact with some specific emotions related to uncertainty and threat (e.g., fear and anxiety) in the reinforcing link between the existential threat and conspiracy theories.

It has also been noted that lower education levels are associated with false beliefs (as well as bias perceptions), suggesting a less skeptical attitude of poorly educated people to fake narratives. Phenomena of functional illiteracy that are too often neglected in our society are fuel for false beliefs, especially as said in such stressful times for individuals.

Moreover, the central role of biased perceptions poses a problem for the effectiveness of scientific communication (especially regarding COVID-19). Research findings show how institutional sources can represent a significant barrier to the spread of misinformation. However, this is not enough because there is a large part of the population that gets informed only by a few, unreliable sources of news and that, in any case, also for psychological reasons, is rather immune to information coming from mainstream media and institutions.

With regard to practical implications, these results shed light on the importance of psychologically supporting those individuals that during long periods of stress and isolation might be more at risk to develop a resigned attitude toward themselves and life events. Fake news related to medical and public health topics can be particularly dangerous for all individuals’ well‐being. Moreover, stress may be an antecedent of beliefs in conspiracy theories^31^. For some individuals, dealing with misinformation could be more difficult under certain circumstances. In sum, we suggest that existing educational interventions should be integrated with psychological ones. The formers are needed to provide tools to recognize possible misleading news, the second ones are crucial to develop a sense of power in mastering not only online information but also one’s own life.

Despite the strengths of the present research, some limitations are worth mentioning. First, the cross-sectional design does not allow to draw conclusions on causal relations between the main variables (i.e., beliefs in misinformation, depression, and bias in perception). Second, other mental health conditions such as anxiety and stress were not assessed, being the association between depression and beliefs in misinformation the focus of the study.

## Supplementary Information

Supplementary Information.

## References

[1] Hofstadter, R. The Paranoid Style in American Politics. Harper’s Magazine, 1964: 77–86.

[2] Brotherton R (2015). Suspicious Minds. Why We Believe Conspiracy Theories.

[3] Ibbetson C. Where do people believe in conspiracy theories? YouGov, 2021.

[4] Uscinski JE, Parent JM (2014). American Conspiracy Theories.

[5] Stein RA, Ometa O, Shetty SP, Katz A, Popitiu MI, Brotherton R (2021). Conspiracy theories in the era of COVID-19. A tale of two pandemics. Int. J. Clin. Pract..

[6] Smith AM, Willroth EC, Gatchpazian A, Shallcross AJ, Feinberg M, Ford BQ (2021). Coping with health threats: The costs and benefits of managing emotions. Psychol. Sci..

[7] O'Connor RC, Wetherall K, Cleare S, McClelland H, Melson AJ, Niedzwiedz CL, Robb KA (2021). Mental health and well-being during the COVID-19 pandemic: longitudinal analyses of adults in the UK COVID-19 Mental Health & Wellbeing study. Br. J. Psychiatry.

[8] Rajkumar RP (2020). COVID-19 and mental health: A review of the existing literature. Asian J. Psychiatr..

[9] Delmastro M, Zamariola G (2020). Depressive symptoms in response to COVID-19 and lockdown: A cross-sectional study on the Italian population. Sci. Rep..

[10] De Coninck D, Frissen T, Matthijs K, d'Haenens L, Lits G, Champagne-Poirier O, Carignan ME, David MD, Pignard-Cheynel N, Salerno S, Généreux M (2021). Beliefs in conspiracy theories and misinformation about COVID-19: comparative perspectives on the role of anxiety, depression and exposure to and trust in information sources. Front Psychol..

[11] Kuhn SAK, Lieb R, Freeman D, Andreou C, Zander-Schellenberg T (2021). Coronavirus conspiracy beliefs in the German-speaking general population: endorsement rates and links to reasoning biases and paranoia. Psychol. Med..

[12] Pennycook G, Rand DG (2021). The psychology of fake news. Trends Cognit. Sci..

[13] Freeman D (2007). Suspicious minds: The psychology of persecutory delusions. Clin. Psychol. Rev..

[14] Freeman D, McManus S, Brugha T, Meltzer H, Jenkins R, Bebbington P (2011). Concomitants of paranoia in the general population. Psychol. Med..

[15] Freeman D, Garety P (2014). Advances in understanding and treating persecutory delusions: A review. Soc. Psychiatry Psychiatr. Epidemiol..

[16] Abalakina-Paap M, Stephan WG, Craig T, Gregory WL (1999). Beliefs in conspiracies. Polit. Psychol..

[17] Cinelli M, Quattrociocchi W, Galeazzi A, Valensise CM, Brugnoli E, Schmidt AL, Zola P, Zollo F, Scala A. The COVID-19 social media infodemic. *Scientific Reports*, 2020: 10(16598). 10.1038/s41598-020-73510-5 PMC7538912 33024152

[18] Brugnoli E, Cinelli M, Quattrociocchi W, Scala A (2019). Recursive patterns in online echo chambers. Sci. Rep..

[19] Cinelli M, De Francisci Morales G, Galeazzi A, Quattrociocchi W, Starnini M. The echo chamber effect on social media, *Proceedings of the National Academy of Sciences*, 2021: 118(9). 10.1073/pnas.2023301118 PMC7936330 33622786

[20] Swami V, Voracek M, Stieger S, Tran US, Furnham A (2014). Analytic thinking reduces belief in conspiracy theories. Cognition.

[21] Deary IJ, Johnson W (2010). Intelligence and education: Causal perceptions drive analytic processes and therefore conclusions. Int. J. Epidemiol..

[22] Delmastro M (2021). On the Measurement of Social Phenomena: A Methodological Approach.

[23] Bavel V (2020). Using social and behavioural science to support COVID-19 pandemic response. Nat. Hum. Behav..

[24] Allcott H, Gentzkow M (2017). Social media and fake news in the 2016 election. J. Econ. Perspect..

[25] Messer S, Angold A, Costello J, Van Kämmen W, Stouthamer-Loeber M (1996). Development of a short questionnaire for use in epidemiological studies of depression in children and adolescents: Factor composition and structure across development. Int. J. Methods Psychiatr. Res..

[26] Thabrew H, Stasiak K, Bavin LM, Frampton C, Merry S (2018). Validation of the Mood and Feelings Questionnaire (MFQ) and Short Mood and Feelings Questionnaire (SMFQ) in New Zealand help-seeking adolescents. Int. J. Methods Psychiatr. Res..

[27] Pennycook G, McPhetres J, Bago B, Rand DG (2020). Predictors of attitudes and misperceptions about COVID-19 in Canada, the UK, and the USA. PsyArXiv.

[28] Douglas KM, Uscinski JE, Sutton RM, Cichocka A, Nefes T, Ang CS, Deravi F (2019). Understanding conspiracy theories. Polit. Psychol..

[29] Schaerer M, Foulk T, du Plessis C, Tu MH, Krishnan S (2021). Just because you're powerless doesn't mean they aren't out to get you: Low power, paranoia, and aggression. Organ. Behav. Hum. Decis. Process..

[30] van Prooijen J-W (2020). An existential threat model of conspiracy theories. Eur. Psychol..

[31] Swami V, Furnham A, Smyth N, Weis L, Lay A, Clow A (2016). Putting the stress on conspiracy theories: Examining associations between psychological stress, anxiety, and belief in conspiracy theories. Personal. Individ. Differ..

