# Supplementary material for: Depression, reduced education, and bias perceptions as risk factors of beliefs in misinformation
Source: Sci Rep. 2022 Sep 30;12:16408. doi: 10.1038/s41598-022-20640-7 (PMC9524309; doi:10.1038/s41598-022-20640-7)
Supplement: Supplementary file 1 — Supplementary Information. [file 41598_2022_20640_MOESM1_ESM.pdf]

Depression, reduced education, and bias perceptions as risk factors of beliefs in misinformation

Marco Delmastro[a,b], Marinella Paciello[c]

[a] Ca' Foscari University, Venice, Italy

[b] Enrico Fermi Research Center, Rome, Italy

[c] Uninettuno Telematic International University, Corso Vittorio Emanuele II, 39, 00186 Rome, Italy

Corresponding Author:

Marco Delmastro

Email: marco.delmastro@cref.it; marco.delmastro@unive.it

ORCID: https://orcid.org/0000-0002-8527-3117

Supplementary Material

Study design

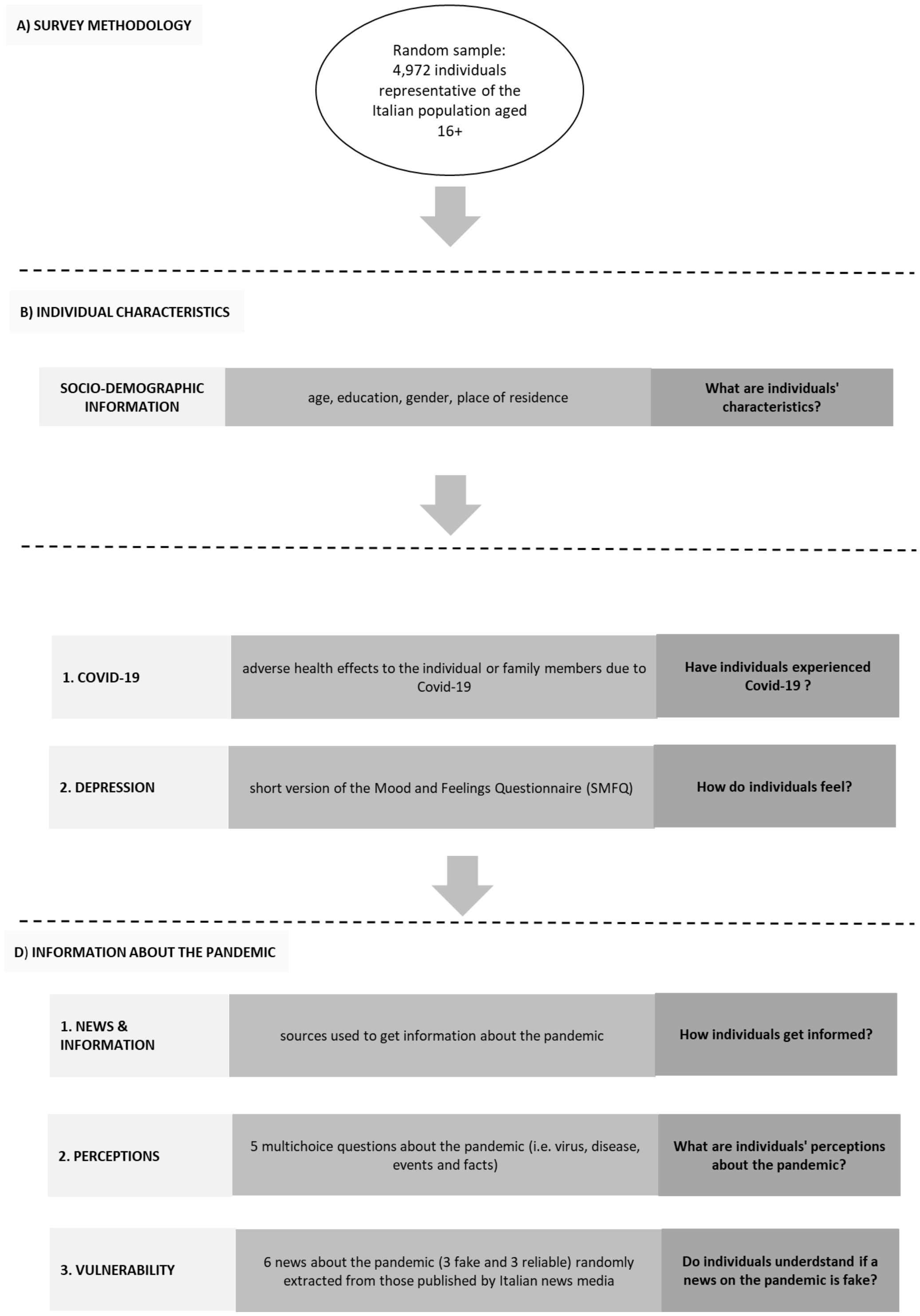

As the first step, all the participants were invited to provide some socio-demographic information about their age, gender, place of residence, and education.

A second step focused on the health status of the participants, in terms of both eventual negative effects (for individuals and/or their families) from the Covid-19 and mental states. The latter was measured by administering the short version of the Mood and Feelings Questionnaire (SMFQ), which tests for depressive symptoms.

After these sections, the interview continued along three blocks of questions.

1. **News sources**. Participants were asked about their news consumption habits in terms of the primary source used to get informed about Covid-19. Participants had 22 different news sources to choose from, which, for the sake of brevity, were grouped into the following 4 categories: traditional sources (i.e., national and local news, in-depth television programs, newspapers, including online newspapers); non-traditional sources (news websites such as VoxNews and Caffeina), algorithmic sources (e.g., Google, Facebook, Twitter, Pinterest), and institutional sources (international, national, regional, and scientific institutions).

2. **Bias in perception.** Participants were interviewed through 5 multiple-choice questions about some of the main topics regarding the COVID-19 pandemic (see Table A.1; the order). The bias in perception has been calculated as a wrong or as a "don't know" answer (i.e., incorrect). For each individual j, the bias in the perception index is therefore given by:

$$BI_j = \frac{\sum_{i=1}^{5} D_{ij}}{5},$$

where: D_i is a dichotomous variable that is 1 if the answer is incorrect and is 0 otherwise.

Table A.1: Topics about Covid-19

**COVID-19 CONTENT**
Prevention
Spread of the virus
Symptoms of Covid-19
Hand cleaning
Disease treatment

3. **Vulnerability to misinformation**. After the questions on perception of the pandemic, participants were randomly presented (drawn from a pool of 12, see Table A.2 for a summary) with a set of 6 news titles reported in an anonymised form (without indication of the news source). At this point, they were asked to indicate a level of trust (or accountability) for each news (i.e., “True/False/I don’t know”). The articles consisted of news on COVID-19 that had actually circulated on Italian media outlets and were chosen among both fake (3 for each individual randomly chosen from 6 fake narratives[1]) and not fake news (3).

[1]

Table A.2: News about Covid-19 (summary)

FAKE NEWS	RELIABLE NEWS
"Two Chinese institutes have indicated that they have two different combinations of medicines that stop the pathogen"	*"Italy, France, Germany and Norway, along with EU summits, announce a global cooperation plan to research a vaccine that counteracts the coronavirus"*
"A report from Lombardy hospitals reports that giving vitamin C to coronavirus patients is critical and is promoting recovery"	*"Antibody treatment of cured people is promising. Preliminary results of this practice seem encouraging but still involve extremely small groups of patients"*
"U.S. coronavirus vaccine ready, able to heal patient within three hours of injection"	*"For paediatricians, the way forward in view of the reopening of schools is the flu vaccination to all children, of any age, and the mandatory tampon for admission to the classroom"*
"The strain of coronavirus that is spreading in China and abroad is a patented virus owned by an entity called The Pirbright Institute, partially funded by the Bill and Melinda Gates Foundation."	*"According to some scientists, tests to detect antibodies to Sars-CoV-2 coronavirus are useful for population-level assessments, but risky for predicting individual immunity"*
"The Italian Red Cross releases an official press release: given the exorbitant prices that Amuchina has reached, here's a recipe for making it at home"	*"With coronavirus emergency, use of disinfectants to protect against infection risk grows. Disinfectant poisoning boom: +65% of requests for help to poison control centers"*
"Accelerated disease was found in coronavirus patients using ibuprofen"	*"7-week-old infant positive for Covid-19 in Parma. It is the first case of virus isolation from an infant"*

Other Tables & Figures

Table A.3: Summary statistics

Variable	Description	N.	Avg.	Std. dv.	Min	Max
Belief in misinformation	1 if the participant believes a false news	14,916	.443	.497	0	1
Bias in perception	Index (0-1) of the number of wrong (or "don't know") answers on the major topics of the Covid-19	4,972	.243	.257	0	1
Gender (female)	1 if female; 0 otherwise	4,972	.511	.499	0	1
Age	Age (number of years)	4,972	48.906	16.444	16	91
Living alone	1 if the individual lives alone; 0 otherwise	4,972	.129	.336	0	1
Education	0 = no education degree (.20%)<br>1= elementary (1.19%)<br>2 = middle school (8.11%)<br>3 = high school (49.84%)<br>4 = graduation (31.46%)<br>5 = master/Ph.D. (9.21%)	4,972	3.387	.824	0	5
Number of sources	Number of news sources used by the individual to get informed about COVID-19	4,877	9.812	.061	0	22
Traditional news outlets	1 if the main source of news about COVID-19 is an established news outlet (i.e., TV, radio, newspaper, online newspaper), 0 otherwise	4,779o	.476	.007	0	1
Non-traditional news outlets	1 if the main source of news about COVID-19 is a non-established online news outlet	4,779o	.009	.001	0	1
Algorithmic news sources	1 if the main source of news about COVID-19 is a search engine or a social media (e.g., Google, Facebook, Twitter), 0 otherwise	4,779o	.098	.004	0	1
Institutional news sources	1 if the main source of news about COVID-19 is an institutional source (e.g., WHO, Italian Ministry of Health, Department of Civil Protection) 0 otherwise	4,779o	.359	.007	0	1
Mood	Short Feeling and Mood Questionnaire Index	4,682	5.473	5.422	0	26
COVID-19	1 if a COVID-19 case in the family of the individual; 0 otherwise	4,972	.0876	.282	0	1

Table A.4: Summary statistics of geographical variables (at the 31st of May 2020)

Variable	N.	Average	Minimum	Maximum
Population of the municipality of residence(*)	2,172	19,715	100	2,761,477
Population of the county of residence	110	550,048	56,938	4,300,000
Population of the region of residence	20	3,013,808	125,666	1.00e+07
Number of COVID-19 cases (county)	110	2,088	22	23,076
Number of COVID-19 deaths (region)	20	1,671	22	16,112

(*) In Italy there are 7,982 municipalities; sample individuals live in 2,172 of them.

Sources: Italian Statistical Office (ISTAT) and Italian Ministry of Health

Figure A.1: Relationship between confirmed Covid-19 cases in the county and probability in believing in misinformation

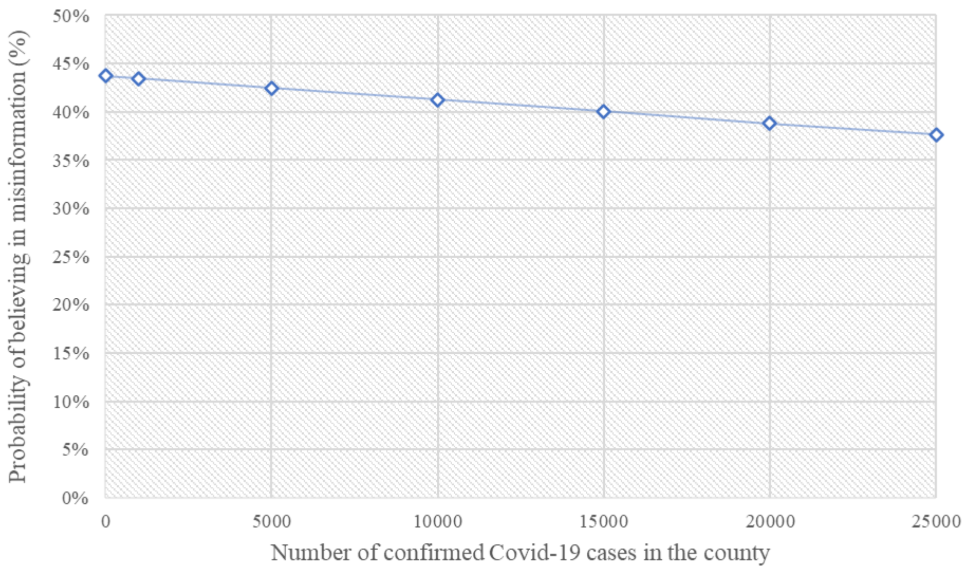

(*) Note: The estimates in the figure refer to a probit panel data model (i.e., Model I of Table 1), with robust standard errors. Pearson correlation coefficient is -0.0303 which is significantly lower than zero.
